# Cervical Spinal Epidural Hematoma After Spinal Anesthesia for Cesarean Section in the Parturient Using Long-Term Low Dose Aspirin

**DOI:** 10.1155/2024/6729275

**Published:** 2024-10-16

**Authors:** Kham Van Vu, Hoang Van Nguyen, Quyen Thi Vu, Thang Toan Nguyen

**Affiliations:** ^1^Center for Anesthesia and Surgical Intensive Care, Bach Mai Hospital, Hanoi, Vietnam; ^2^Department of Anesthesia and Intensive Care, Hanoi Medical University, Hanoi, Vietnam

**Keywords:** cervical spinal epidural hematoma, low-dose aspirin, surgical intervention

## Abstract

Spinal epidural hematoma (SEDH) is a rare but serious complication associated with spinal anesthesia (SA). We present an unusual case of cervical SEDH occurring 24 h after a lumbar puncture for a cesarean section. The patient, who was on low-dose aspirin due to preeclampsia, initially exhibited neurological symptoms resembling a stroke. Despite a normal magnetic resonance imaging (MRI) of the brain, further investigations revealed a SEDH located between the C3 and T1 segments, well beyond the L3-L4 puncture site. Although coagulation tests were normal, this case underscores the potential risk of low-dose aspirin in affecting platelet function, which may contribute to SEDH development. It also emphasizes the importance of considering spinal MRI when neurological symptoms arise after SA, even if initial cranial MRI results are normal. She underwent emergency C3–T1 laminectomy through a dorsal midline approach. Her motor, sensory, and sphincter functions fully recovered at follow-up.

## 1. Introduction

Spinal epidural hematoma (SEDH) is rare but a highly severe complication related to epidural anesthesia (EA) or spinal anesthesia (SA). It can occur at the level where the needle is inserted for SA, or even at the highest level reached by the catheter, persisting after removal. Our research indicates that there are few reported cases of SEDH occurring remote from the puncture site for SA [1, 2], and there is only published report on cervical epidural hematoma following SA [3]. Complicating the diagnosis, the patient's initial clinical presentation resembled a stroke, leading to a delayed and challenging diagnosis. SEDH after SA is believed to result from the rupture of venous plexuses in the spine, which is more common in individuals of advanced age, female gender, those with spinal abnormalities, and coagulation disorders [4]. According to the American Society of Regional Anesthesia, low-dose aspirin therapy is not a contraindication for regional techniques and is generally considered safe for SA.

## 2. Case Report

A 39-year-old woman (weight: 60 kg, height: 158 cm, and body mass index [BMI]: 24), previously healthy (the American Society of Anesthesiologists [ASA] physical status classification: 1) and pregnant with in vitro fertilization twins, was prescribed 81 mg of aspirin starting at Week 14 due to a high risk of preeclampsia (multiple gestations and advanced maternal age). She underwent a scheduled cesarean section at 37 weeks because of the preeclampsia and twins. Prior to surgery, her basic coagulation tests include platelet count, and coagulation tests were normal (Table 1). The patient was transferred to the operating room and connected to monitoring devices, with all vital signs remaining within normal physiological limits. A lumbar puncture at the L3-L4 interspace was successfully performed on the first attempt using a Quincke G25 needle, without complications. The surgery proceeded smoothly, with stable hemodynamic parameters and no need for vasoactive drugs. She delivered healthy twins, with Apgar scores of 9 at 1 min and 10 at 5 min. Postoperatively, she was monitored for 6 h without complications in a postoperative room.

However, 24 h after surgery, she developed a severe headache and gradually increasing muscle weakness on the right side of her body, with muscle strength measured at 2/5 in the right upper and lower limbs. A brain MRI showed no abnormalities, while laboratory tests indicated a platelet count of 114 × 10^9^/L (G/L) and normal clotting function. Initially diagnosed with transient cerebral ischemia, she received conservative treatment, but her condition showed poor improvement.

**Figure 1:**
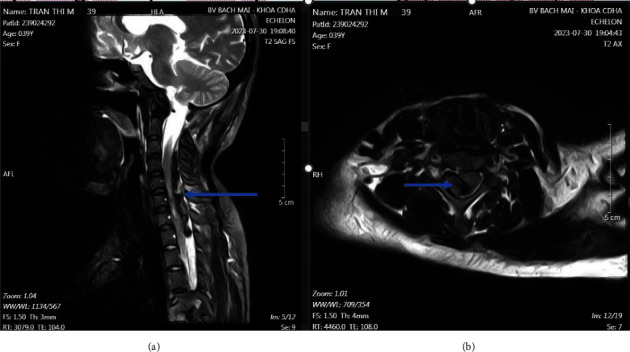
(a) Postoperative T2–weighted sagittal image showing evidence of epidural hematoma in the C3-T1 segment (marked with an arrow); (b) postoperative T2–weighted axial image showing evidence of epidural hematoma (marked with an arrow).

By 48 h postsurgery, her condition had deteriorated and left-sided paralysis had begun. She was transferred to our hospital with a Glasgow Coma Scale score of 15, stable blood pressure, and complete muscle paralysis on the right side, with muscle strength measured at 0/5. She also experienced external anal sphincter dysfunction. A spinal MRI was performed immediately at the 48th hour revealed an epidural hematoma from C3 to T1, with no vascular malformations or other damage detected (Figure 1). She underwent an emergency C3–T1 laminectomy via a dorsal midline approach under general anesthesia 50 h after the cesarean section. Although her initial coagulation tests were normal, platelet aggregation tests on Day 6 after stopping aspirin showed significant reductions (29% with ADP, 32% with collagen, and 1% with arachidonic acid), returning to normal by Day 12 (Table 2).

She was treated in the surgical intensive care unit and rehabilitation department. Remarkably, her motor, sensory, and external anal sphincter dysfunction fully recovered after 2 months.

## 3. Discussion

After epidural anesthetics, estimates of the rate of the epidural hematoma range from one in 150,000 to one in 2700. In patients with abnormal coagulation, the rate may be as low as one in 315 epidural anesthetics. After spinal anesthetics, the rate is estimated to be one in 220,000 patients [5].

Aspirin has a broad spectrum of pharmacological effects, including analgesic, antipyretic, antiplatelet, and vasomotor properties [6]. Although the precise mechanism by which aspirin influences preeclampsia is not completely understood, its antithrombotic and vasodilatory effects, along with the restoration of the balance between thromboxane and prostacyclin, are believed to significantly enhance placentation. Furthermore, its anti-inflammatory properties may also play a role. The American Society of Regional Anesthesia recommends that low-dose aspirin therapy is not a contraindication for neuraxial anesthesia, considering it safe when platelet counts exceed 75 G/L [7]. A count below 50 G/L is typically seen as a contraindication, while those between 50 and 75 (G/L) require individual assessment based on patient-specific risks and coagulation tests. However, we must pay attention to platelet function after aspirin use. According to the study by Jeske M. Bij de Weg et al. [8], aspirin 80 mg significantly inhibits platelet function in laboratory tests during pregnancy when compared to a placebo, this inhibition is observed similarly in both the second and third trimesters of pregnancy. In our patient, platelet aggregation remained reduced on the sixth day after stopping aspirin, returning to normal by the 12th day, potentially contributing to the risk of SEDH. Moreover, there are documented cases of spontaneous epidural hematoma following low-dose aspirin use, unrelated to SA and with no other identified risk factors. This underscores the risk associated with low-dose aspirin in our case [9–11]. We emphasize the importance of exercising caution when performing neuraxial anesthesia on patients using low-dose aspirin. It is crucial to strike a balance between benefits and risks and to closely monitor postoperative recovery to promptly detect and manage any complications.

SEDH can occur at the site of needle insertion for SA, but few cases have been reported where SEDH occurred remotely from the puncture site [1, 3]. The mechanism for such remote hematomas remains speculative; one possibility is that during pregnancy, the gravid uterus exerts pressure on the inferior vena cava (IVC), leading to chronic engorgement of the epidural venous plexus. Positioning the patient in the lateral decubitus position for SA may stretch these engorged veins, potentially triggering a remote bleed in the thoracic region. The sudden decompression of the IVC following delivery may further exacerbate this stretching, resulting in SEDH at any point along the spine [1].

An important aspect of our case is the initial clinical presentation, which resembled a stroke, complicating the diagnosis. The patient experienced headache and unilateral right-sided muscle weakness, indicative of cerebral infarction or intracranial hemorrhage. Despite normal cranial MRI results, it was not until left-sided weakness and sphincter dysfunction developed that a spinal MRI was performed, revealing the epidural hematoma. The occurrence of intracranial hematomas after SA for cesarean sections has been documented with similar clinical symptoms and timing [12]. Thus, even with normal cranial MRI findings, prompt spinal MRI is crucial to rule out other complications.

Regarding treatment, there is ongoing debate about whether to pursue surgical or conservative management. Immediate surgical decompression is the preferred approach for most patients; however, conservative management may be appropriate in certain cases [13]. Neurological improvement and mild neurological deficit without progression are good indications for a conservative approach [14]. The presence of significant coagulopathy and/or anticipated risks associated with surgical intervention may also serve as relative indications for conservative treatment [15]. In our case, rapid progression of symptoms necessitated emergency surgery. The patient's favorable recovery, achieved within 50 h of symptom onset, supports our decision for immediate intervention.

## 4. Conclusion

Our case suggests that low-dose aspirin can significantly impair platelet aggregation, potentially contributing to the development of SEDH. We should proceed with caution when administering neuraxial anesthesia to patients taking low-dose aspirin, notwithstanding the current guidelines permitting this practice. Notably, SEDH after SA can manifest later and at a distance from the needle insertion site. Therefore, any patient who does not recover from a neuraxial block within the expected timeframe or who develops new neurological symptoms should undergo a neuro-MRI, encompassing both the brain and spinal cord. If a spinal hematoma is identified, particularly in cases of large hematomas with compression evident on MRI and rapidly progressing clinical symptoms, urgent surgical intervention is essential and can lead to favorable outcomes.

## Figures and Tables

**Table 1:** Platelet count and coagulation tests Day 0th and Day 2.

	Day 0	Day 2
PT (%)/INR	102/0.98	91/1.04
APTTs (second)	29	27
Fibrinogen (g/L)	4.59	4.8
Platelet count (G/L)	170	114

Abbreviations: APTTs: activated partial thromboplastin time; INR: international normalized ratio; PT: prothrombin time.

**Table 2:** Platelet aggregation tests on Day 6 and Day 12.

Platelet aggregation tests	Day 6 (%)	Day 12 (%)	References (%)
ADP	**29**	67	60–75
Collagen	**32**	60	60–75
Arachidonic	**1**	80	74–99
Ristocetin	82	75	60–80

*Note:* Bold values indicate that they are lower than the normal range.

Abbreviation: ADP: adenosine diphosphate.

## Data Availability

Data sharing is not applicable to this article as no datasets were generated or analyzed during the current study.
